# Disparate Intent-to-Treat Outcomes for Pediatric Liver Transplantation Based on Indication

**DOI:** 10.1155/2023/2859384

**Published:** 2023-03-02

**Authors:** Anna Lang, Cameron Goff, Ashley Montgomery, Jake Lynn, Spoorthi Kamepalli, John Goss, Abbas Rana

**Affiliations:** ^1^Department of Student Affairs, Baylor College of Medicine, Houston, USA; ^2^Division of Abdominal Transplantation, Michael E DeBakey Department of General Surgery, Baylor College of Medicine, Houston, USA

## Abstract

**Background:**

The impact of indication for pediatric liver transplantation on waitlist and post-transplant mortality outcomes is well known, but the impact on intent-to-treat outcomes has not been investigated. Intent-to-treat survival analysis is important in this study because it is more comprehensive, combining the transplant outcomes of waitlist mortality, post-transplant mortality, and transplant rate into a single metric to elucidate any disparities in outcomes based on indication.

**Methods:**

Cox regression was used to analyze factors impacting survival in 8,002 children listed for liver transplant in the UNOS database between 2006 and 2016. The Kaplan–Meier method and log-rank test were used to assess differences in waitlist, post-transplant, and intent-to-treat mortality among the top 5 indications of biliary atresia, acute hepatic necrosis, metabolic disorders, hepatoblastoma, and autoimmune cirrhosis.

**Results:**

When compared to the reference group of biliary atresia, multivariate analyses showed that every indication was associated with inferior intent-to-treat outcomes except for metabolic disorders. Hepatoblastoma (hazard ratio (HR): 3.73), autoimmune cirrhosis (HR: 1.86), and AHN (HR: 1.77) were associated with significantly increased intent-to-treat mortality. Hepatoblastoma was also associated with increased post-transplant mortality (HR: 3.77) and was the only indication significantly associated with increased waitlist mortality (HR: 6.43).

**Conclusion:**

Significant disparity exists across all indications with respect to an increased intent-to-treat mortality, along with an increased post-transplant and waitlist mortality, when compared to the biliary atresia reference group. If further studies validate these findings, a reexamination of the equitable distribution of allografts for transplant may be warranted as well as a focus on disparities in survival after transplant.

## 1. Introduction

Waitlist and post-transplant outcomes and access have steadily improved for pediatric liver transplantation over the past two decades. On the other hand, the prevalence of indications has remained relatively constant [1, 2]. Regardless of indication, listed liver transplant candidates are prioritized based on the PELD (pediatric end-stage liver disease) scoring system, a system which is designed to predict waitlist mortality. This current allocation system can also give special priority to patients outside the parameters of the PELD score by assigning a “status 1” priority that is designated for critically ill patients and by appealing to regional review boards on a case-by-case basis [1, 3, 4]. However, this PELD/exception system does not account for post-transplant mortality, which together with waitlist mortality (in an intent-to-treat analysis) would provide a more comprehensive assessment of the variation in outcomes in liver transplantation [5]. With respect to transplant indication, cholestatic disease, such as biliary atresia, has steadily remained the top indication for pediatric liver transplant [2, 6]. Accordingly, in pediatrics, the impact of a biliary atresia diagnosis on waitlist and post-transplant outcomes has been well studied, but these individual metrics alone are incomplete in describing the mortality risk that is associated with biliary atresia and other top indications for transplant [7, 8]. In this study, the more composite, comprehensive nature of an intent-to-treat analysis would provide additional insight into the impact of transplant indication on overall mortality.

An intent-to-treat analysis measures survival from listing until death regardless of whether a transplant is received. In this study, an intent-to-treat analysis is employed as the metric to assess equity among outcomes because it is more representative of the impact of transplant indication on outcomes than individual metrics alone. Individual metrics, such as waitlist mortality and post-transplant mortality, are and have been crucial in tracking progress in transplantation, but each individually falls short of representing the true mortality risk from an intent-to-treat perspective. It is becoming increasingly important to understand how waitlist mortality and post-transplant mortality can influence each other across time; this temporal relationship is better illustrated in an intent-to-treat analysis. Specifically, because intent-to-treat is a function of waitlist mortality, post-transplant mortality, and transplant rate, it provides a more comprehensive perspective in assessing the outcome equity between indications in pediatric liver transplantation [5].

The primary aim of this study is to examine differences in intent-to-treat mortality among the top indications for pediatric liver transplantation in order to describe possible disparate outcomes between indications for transplant. By investigating the impact of indication on intent-to-treat mortality, this study will provide more insight into characterizing each transplant indication based on the more comprehensive mortality risk measurement that an intent-to-treat analysis provides.

## 2. Methods

### 2.1. Study Population

This study is a retrospective analysis of the UNOS deidentified patient-level data of all pediatric patients listed for liver transplantation between January 1, 2006, and December 31, 2016. Patients were identified in the Organ Procurement and Transplantation Network (OPTN) registry, which includes data on all solid organ transplants in the United States. All patients younger than 18 years who were listed for transplant were included (*n* = 8,002). Candidate characteristics were reported at the time of listing. Candidates who were undergoing simultaneous solid organ transplants (including heart, intestine, lung, kidney, or pancreas) were excluded (*n* = 4). Patients listed for a retransplant were included in the analysis unless they did not meet the other criteria defined here.

### 2.2. Classifying Disease Indication

Diagnosis groups are described in Table 1; they represent the top 5 indications for pediatric liver transplantation based on data from 2006 to 2016. These groups, in descending order of frequency, are as follows: biliary atresia, acute hepatic necrosis (AHN), metabolic disorders, hepatoblastoma, and autoimmune cirrhosis. Biliary atresia served as the reference group. Different variations of the same general diagnosis category were grouped together; for example, patients listed with alpha-1-antitrypsin deficiency would be placed in the same indication category (metabolic disorders) as patients listed with Wilson's disease.

### 2.3. Outcome Analysis

Mortality analyses as well as descriptive statistics were performed in Stata 17 (Stata Corp) to characterize each disease group. Continuous variables were reported as mean ± standard deviation and compared using Student's *t*-test. Results were considered significant at a *p* value of <0.05. The primary outcome was mortality, assessed as intent-to-treat mortality (from listing until death, regardless of transplantation status), waitlist mortality (from listing until death or removal from the waitlist, for patients who did not receive a transplant), and post-transplant mortality (from transplant until death). Removal from the transplant waiting list was also categorized as death if designated as removal for being too sick to transplant but was otherwise considered a last known follow-up date. Mortality analyses consisted of standard Kaplan–Meier curves and log-rank tests. Intent-to-treat mortality curves were measured from time of listing to time of death or last known follow-up, regardless of transplantation status. Waitlist mortality curves were measured from time of listing to time of death or removal from the waiting list. Post-transplant mortality curves were measured from time of transplantation to time of death or last known follow-up. Statistical significance was evaluated by univariate and multivariate cox regression tests. Covariables found to be significant in univariable regression (defined as *p* < 0.05) were included in multivariable regression, in addition to the top five indications for transplant.

### 2.4. Risk Factors

Recipient risk factors considered in this analysis are listed in Table 2. Continuous variables were categorized using clinically relevant groupings. Candidate age, height, and weight are represented as the calculated values for that candidate at listing. Gender/sex, ethnicity/race, and insurance payment type are represented as the candidate was described in listing documentation.

### 2.5. Percentage Transplanted

The percentage of all listed patients who received transplant within 1 year was calculated for each transplant indication. Chi-squared tests were used to compare each percentage to the reference group of all other transplant indications. The total percentage of patients who received transplant and mean wait time to transplant were also calculated for each transplant indication.

## 3. Results

### 3.1. Study Population

The study population included 8,002 patients. Of the study population, 2,209 (27.6%) were diagnosed with biliary atresia, 1,056 (13.2%) were diagnosed with AHN, 899 (11.2%) were diagnosed with a metabolic disorder, 450 (5.6%) were diagnosed with hepatoblastoma, and 206 (2.6%) were diagnosed with autoimmune cirrhosis. Mean follow-up time was 6.07 years for biliary atresia patients, 4.25 years for AHN patients, 5.62 years for metabolic disorder patients, 5.66 years for hepatoblastoma patients, and 3.59 years for autoimmune cirrhosis patients. Demographic and clinical characteristics for recipients stratified by transplant indication are summarized in Table 2.

### 3.2. Intent-to-Treat Mortality

**Figure 1:**
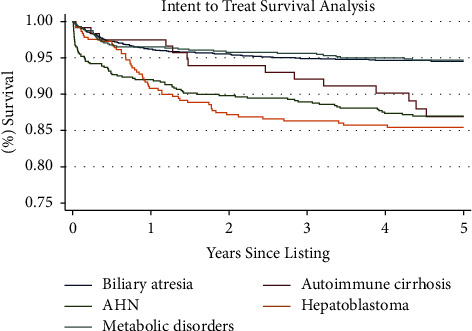
Intent-to-treat survival analysis. Kaplan–Meier survival function over 5 years for patient survival in pediatric patients listed for liver transplant from listing until death, regardless of transplantation status, by indication.

Table 3 lists the risk factors that were included in multivariable analysis; variables found significant in multivariable analysis (*p* < 0.05) can be seen in bold. All indications except for metabolic disorders were found to be significant. Hepatoblastoma (with a 95% confidence interval (CI) of 2.61–5.26), autoimmune cirrhosis (CI: 1.09–3.23), and AHN (CI: 1.27–2.49) were associated with an increased intent-to-treat mortality. The corresponding Kaplan–Meier curve can be seen in Figure 1, which illustrates that intent-to-treat mortality was significantly higher in the hepatoblastoma subgroup compared to other disease groups.

In addition to disease indication, several other factors affecting intent-to-treat mortality were identified. Age at listing (CI: 1.01–1.04), life support status (CI: 1.84–3.08), dialysis status (CI: 1.40–3.30), and public insurance (CI: 1.06–1.90) were associated with an increased intent-to-treat mortality. Initial albumin levels (0.68–0.86) were associated with a decreased intent-to-treat mortality.

### 3.3. Waitlist Mortality

**Figure 2:**
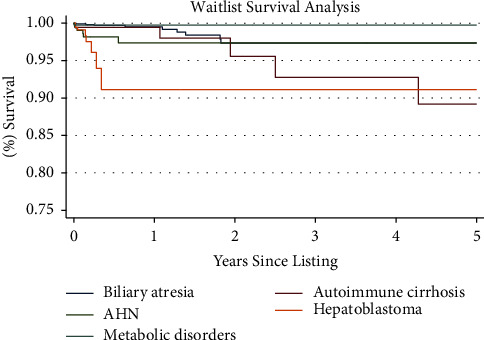
Waitlist survival analysis. Kaplan–Meier survival function over 5 years for patient survival in pediatric patients listed for liver transplant from listing until death or removal from the waitlist, without undergoing transplantation, by indication.

Table 4 lists the risk factors that were included in multivariable analysis; variables found significant in multivariable analysis (*p* < 0.05) can be seen in in bold. The corresponding Kaplan–Meier curve can be seen in Figure 2. Mortality on the waiting list (that is, without transplant) varied by indication. Multivariable analysis showed that of the top 5 indications for transplant, only hepatoblastoma was associated with an increased waitlist mortality, with a 95% confidence interval of 2.47–18.84.

Additionally, age at listing (CI: 1.01–1.08), life support status (CI: 1.28–4.71), the rank of status 1 (CI: 1.77–6.10), and public insurance (CI: 1.20–2.65) were also associated with an increased waitlist mortality.

### 3.4. Post-Transplant Mortality

**Figure 3:**
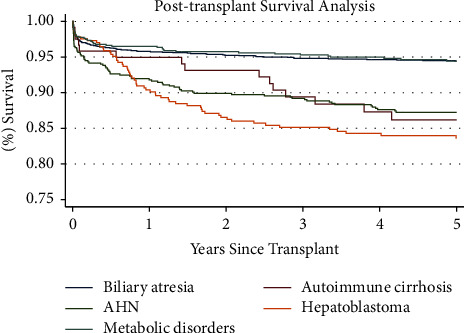
Post-transplant survival analysis. Kaplan–Meier survival function over 5 years for patient survival in pediatric patients who underwent liver transplant from transplantation until death, by indication.

Table 5 lists the risk factors that were included in multivariable analysis; variables found significant in multivariable analysis (*p* < 0.05) can be seen in in bold. The corresponding Kaplan–Meier curve can be seen in Figure 3. The multivariable trends observed in the post-transplant mortality analysis followed a similar pattern as those observed in the intent-to-treat mortality analysis. All indications except for metabolic disorders were found to be significant. Hepatoblastoma (CI: 2.69–5.32), autoimmune cirrhosis (CI: 1.13–3.28), and AHN (CI: 1.19–2.35) were associated with an increased post-transplant mortality.

Similar factors identified as significant in the intent-to-treat multivariable analysis were likewise significant in the post-transplant multivariable analysis, with the addition of two regions also being significant. Age at listing (CI: 1.01–1.04), life support status (CI: 1.77–2.96), dialysis status (CI: 1.31–3.15), and public insurance (CI: 1.04–1.90) were associated with an increased post-transplant mortality. Region 6 listing (in AK, HI, ID, MT, OR, and WA) (CI: 0.25–0.96), region 5 listing (in CA, NV, AZ, UT, and NM) (CI: 0.63–1.00), and initial albumin levels (0.78–0.89) were associated with a decreased post-transplant mortality.

### 3.5. Percentage Transplanted

The percentage of all listed patients who received transplants within 1 year was calculated for each transplant indication. Variables found significant in chi-squared analysis (*p* < 0.05) can be seen in Table 6 in bold, along with mean wait times among patients who received a transplant. The hepatoblastoma diagnosis group had the most patients transplanted in the first year (85.1%). Significantly more hepatoblastoma patients were transplanted within the first year than autoimmune cirrhosis patients, who only had 47.6% transplanted within the first year. Mean wait time, from waitlist until transplant, also varied across indication, from the shortest mean wait time of 23.6 days for hepatoblastoma patients to the longest mean wait time of 386.8 days for autoimmune cirrhosis patients.

## 4. Discussion

This study analyzes pediatric liver transplant intent-to-treat outcomes from the perspective of the most common transplant indications. Of the total population of 8,002 patients, 4,820 patients (60.23%) had an indication of biliary atresia, AHN, metabolic disorders, hepatoblastoma, and autoimmune cirrhosis. Waitlist and post-transplant mortality are well studied in general terms in liver transplantation, but the impact of indication on the more comprehensive metric of intent-to-treat mortality is yet to be fully investigated [9, 10]. In this study, multivariable analysis was performed on clinically relevant variables from 8,002 patients from the UNOS database in order to describe the association between indication and intent-to-treat outcomes throughout the transplantation process and to describe potential disparities. Multivariate analysis showed that every indication was associated with significantly inferior outcomes, particularly an increased intent-to-treat mortality, when compared to the reference group of biliary atresia. Specifically, hepatoblastoma was associated with the highest intent-to-treat (HR: 3.73), waitlist (HR: 6.43), and post-transplant (HR: 3.77) out of the selected indications.

The pediatric end-stage liver disease (PELD) scoring system was implemented in 2002 and designed to equitably distribute organs for transplant based on the risk of 90-day pretransplant death [3, 9, 11–13]. Currently, the PELD system combines a series of measurable, objective criteria, including serum bilirubin, creatinine, and INR, to calculate a numerical score that can be used to assign priority for transplant. Additional special priority, such as the assignment of status 1A or 1B, is also awarded to risk factors that are indicative of imminent mortality that cannot be reflected by PELD's clinical metrics alone [14, 15]. For example, status 1B is often applied to patients with unresectable hepatoblastoma. In our study population, hepatoblastoma had the highest proportion of status 1B patients at 36.2%. Because of hepatoblastoma's aggressive and metastatic nature, prompt liver transplant is the only available treatment for those whose hepatoblastoma is deemed unresectable [16].

Of the indications analyzed, hepatoblastoma was associated with increased mortality across all three outcome metrics. In particular, hepatoblastoma had the highest intent-to-treat mortality out of all indications analyzed (HR: 3.73). However, hepatoblastoma also had the most patients transplanted within the first year of listing out of any indication studied (85.1%) and the corresponding shortest mean wait time (23.6 days). Children who have hepatoblastoma are associated with increased waitlist, post-transplant, and intent-to-treat mortality primarily because of the pointedly harmful pathogenesis of their diagnosis. Because of this aggressive nature, it follows that hepatoblastoma patients must then have the shortest waitlist times if they are to have the best chances of survival [17].

Similarly, autoimmune cirrhosis was associated with increased mortality across intent-to-treat (HR: 1.86) and post-transplant (HR: 1.89) outcomes. AHN, or acute hepatic necrosis, was also associated with increased intent-to-treat mortality (HR: 1.77) and post-transplant mortality (HR: 1.65). Both indications do not have a significant difference from the reference group biliary atresia in waitlist mortality, although both do have a significant difference from the reference group in post-transplant mortality. This divergence in statistical significance between waitlist and post-transplant mortality highlights the importance of the utilization of intent-to-treat mortality in this study. With respect to these two indications of autoimmune cirrhosis and AHN, intent-to-treat mortality is a single yet comprehensive measure of transplant outcome that is better able to encompass the impact of transplant indication on mortality better than the individual metrics of waitlist or post-transplant mortality alone.

Because it has been consistently the top indication for pediatric liver transplantation, biliary atresia has been thoroughly examined in pediatric transplant literature [6, 18]. Most studies concerning biliary atresia focus on differing clinical variables within the diagnosis itself with respect to waitlist and post-transplant outcomes, without comprehensively evaluating the overall mortality of biliary atresia in an intent-to-treat analysis. In our study, biliary atresia was used as the reference group. As shown by the relative increase in intent-to-treat mortality of all other indications, patients with biliary atresia have favorable survival intent-to-treat outcomes. Even though they have only 51.0% of patients transplanted within 1 year, biliary atresia patients still have a favorable intent-to-treat morality. So, despite relatively longer wait times, the biliary atresia patients still have relatively positive intent-to-treat transplant outcomes.

Prior to this study, there had not been a comprehensive evaluation of the impact of pediatric liver transplant indication on intent-to-treat outcomes. As a composite function of waitlist mortality, post-transplant mortality, and transplant rate, this intent-to-treat analysis aims to describe and quantify differences in outcomes between transplant indications. In the context of the intent-to-treat results produced from this analysis based on indication, future research should validate these findings and then analyze how the PELD/exception organ allocation system can adjust or add exceptions to attempt to compensate for these outcome disparities, perhaps also with a focus on disparities in survival after transplant.

### 4.1. Limitations

Because the scope of this study included only pediatric patients undergoing a single organ liver transplant, the results are not generalizable to children undergoing a multiorgan transplant. Retransplantation candidates were included here because retransplantation is increasing in frequency, although still relatively uncommon. While indications for retransplantation can be categorized in the same groups as indications for primary transplant, they are still a unique subpopulation with an already altered and distinct clinical picture. Because our study is from a large database, it is limited by the lack of granular clinical data and fine details concerning each indication beyond the standardized database variables. Additionally, the large nature of the database used makes it intrinsically prone to errors in data input, but this should not affect results due to the substantial size of cohort of study.

## 5. Conclusion

Intent-to-treat mortality is a more comprehensive and representative outcome metric that measures survival from listing until death, regardless of whether a transplant is received. Intent-to-treat outcomes evaluated in the context of transplant indication illustrate the disparities across mortality outcomes of different indications, specifically with respect to intent-to-treat mortality. When compared to biliary atresia, the indications of hepatoblastoma, autoimmune cirrhosis, and AHN had a significantly increased intent-to-treat mortality, with hepatoblastoma being the highest.

## Figures and Tables

**Table 1:** Transplant indication.

Indication type	Diagnoses included
Biliary atresia	Extrahepatic biliary atresia
AHN	Drug, type A, type B, type non-A non-B, type C, type D, type D, type B and C, type B and D, acute viral infection, autoimmune hepatitis
Metabolic disorders	Alpha-1-antitrypsin deficiency, Wilson's disease, hemochromatosis, glycogen storage disease (I, IV), hyperlipidemia II, homozygous hypercholesterolemia, tyrosinemia, primary oxalosis, maple syrup urine disease, urea cycle defects
Hepatoblastoma	Hepatoblastoma
Autoimmune cirrhosis	Autoimmune cirrhosis

**Table 2:** Demographics and clinical characteristics by indication.

Variable	Indication					
	Biliary atresia	Hepatoblastoma	Autoimmune cirrhosis	AHN	Metabolic disorders	Percent entry missing
Age (mean)	2.1	2.9	14.7	7.9	6.0	0.0
Male sex (%)	39.6	63.3	32.5	47.3	55.5	0.0
Race and ethnicity (%)
White	48.6	56.7	45.1	42.5	65.0	0.0
Black	17.1	8.4	31.6	16.4	7.6	0.0
Hispanic	21.5	25.1	18.4	32.6	20.7	0.0
Asian American	8.1	5.8	1.9	4.5	5.3	0.0
Other	4.7	4.0	2.9	4.0	1.4	0.0
Height (cm) (mean)	80.7	90.3	157.1	122.5	107.9	28.2
Weight (kg) (mean)	12.8	14.4	58.9	35.4	26.9	28.5
Life support (%)	3.8	4.0	1.9	26.6	4.3	0.0
Dialysis (%)	1.0	1.1	1.0	4.1	2.2	0.0
Insurance (%)
Private	44.5	46.8	55.3	48.2	48.8	0.0
Public	45.1	41.8	38.8	38.9	34.6	0.0
Other form of payment	10.5	11.3	5.8	12.9	16.6	0.0
Status 1 (%)	3.8	38.9	1.9	68.9	11.8	0.0
Status 1A	2.7	2.7	1.5	67.7	10.1	
Status 1B	1.0	36.2	0.5	1.1	1.7	
Exception submission (%)	50.2	51.0	42.3	6.17	75.4	0.0
Inactive (%)	1.7	1.3	2.9	0.2	0.9	0.0
US citizen (%)	97.4	97.1	99.0	96.8	91.2	0.0
Region (%)
CT, ME, MA, NH, RI	4.4	4.4	1.9	2.7	4.4	0.0
DE, DC, MD, NJ, PA, N. VA, WV	13.5	14.2	12.6	8.9	23.2	0.0
AL, AR, FL, GA, LA, MS, PR	10.4	10.0	19.9	11.3	8.8	0.0
OK, TX	12.3	10.0	17.0	14.3	10.5	0.0
AZ, CA, NV, NM, UT	20.1	23.1	18.4	27.4	17.2	0.0
AK, HI, ID, MT, OR, WA	3.0	2.0	1.9	2.9	2.7	0.0
IL, MN, ND, SD, WI	8.8	6.7	5.3	8.6	12.1	0.0
CO, IA, KS, MO, NE, WY	7.5	9.3	5.3	6.6	6.7	0.0
NY, VT	5.2	5.1	4.4	4.1	5.3	0.0
IN, MI, OH	8.6	10.9	4.4	7.3	6.5	0.0
KY, NC, SC, TN, VA	6.2	4.2	8.7	6.0	2.6	0.0
Blood type (%)
A	32.2	32.9	31.6	30.6	31.0	0.0
B	14.1	11.1	15.0	11.7	14.2	0.0
AB	4.3	3.8	2.9	3.7	4.1	0.0
O	49.4	52.2	50.0	54.0	50.6	0.0
Graft type
Whole cadaveric	42.2	52.0	50.4	38.7	56.8	71.3
Technical variant cadaveric	39.7	33.1	8.7	20.9	25.8	71.3
Live donation	14.0	4.4	4.3	5.3	5.0	71.5

**Table 3:** Multivariable analysis: intent-to-treat mortality.

Variable	Intent-to-treat mortality		
	*P* value	95% CI	Hazard ratio
Age	**0**	**1.01**–**1.04**	**1.02**
Indication			
Hepatoblastoma	**0**	**2.61**–**5.26**	**3.71**
Autoimmune cirrhosis	**0.02**	**1.09**–**3.23**	**1.87**
AHN	**0**	**1.27**–**2.49**	**1.78**
Metabolic disorders	0.51	0.78–1.63	1.13
Other	**0**	**1.68**–**2.74**	**2.15**
Race and ethnicity			
White	0.15	0.71–1.05	0.86
Black	0.97	0.78–1.25	0.99
Height (cm)			
Donor-recipient height difference over 60 cm	0.99	0.80–1.23	0.99
Life support	**0**	**1.84**–**3.08**	**2.38**
Dialysis	**0**	**1.40**–**3.30**	**2.15**
Insurance			
Private	0.92	0.72–1.32	0.98
Public	**0.02**	**1.06**–**1.90**	**1.42**
Status 1	0.67	0.74–1.21	0.94
Region			
AK, HI, ID, MT, OR, WA	0.05	0.26–1.00	0.51
Initial laboratory values			
Bilirubin	0.14	0.99–1.01	1.00
INR	0.50	0.96–1.07	1.01
Creatinine	0.12	0.93–1.77	1.28
Albumin	**0**	**0.68**–**0.86**	**0.76**

**Table 4:** Multivariable analysis: waitlist mortality.

Variable	Waitlist mortality		
	*P* value	95% CI	Hazard ratio
Age	**0**	**1.01**–**1.08**	**1.04**
Indication			
Hepatoblastoma	**0**	**2.47**–**18.84**	**6.82**
Autoimmune cirrhosis	0.20	0.69–5.91	2.02
AHN	0.99	0.35–2.83	1.00
Metabolic disorders	0.30	0.09–2.04	0.44
Other	**0**	**1.76**–**6.88**	**3.48**
Race and ethnicity			
Black	0.37	0.77–1.94	1.23
Life support	**0**	**1.28**–**4.71**	**2.45**
Insurance			
Public	**0**	**1.20**–**2.65**	**1.78**
Status 1	**0**	**1.77**–**6.10**	**3.28**
Region			
AR, LA, MS, AL, GA, FL	0.15	0.85–2.75	1.53
Initial laboratory values			
INR	0.06	0.99–1.16	1.07
Albumin	0.14	0.62–1.06	0.81

**Table 5:** Multivariable analysis: post-transplant mortality.

Variable	Post-transplant mortality		
	*P* value	95% CI	Hazard ratio
Age	**0**	**1.01**–**1.04**	**1.03**
Indication			
Hepatoblastoma	**0**	**2.69**–**5.32**	**3.78**
Autoimmune cirrhosis	**0.02**	**1.13**–**3.28**	**1.92**
AHN	**0**	**1.19**–**2.35**	**1.68**
Metabolic disorders	0.70	0.74–1.55	1.07
Other	**0**	**1.58**–**2.59**	**2.02**
Race and ethnicity			
White	0.10	0.69–1.03	0.84
Black	0.66	0.74–1.20	0.94
Height (cm)			
Donor-recipient height difference over 60 cm	0.96	0.80–1.23	0.99
Life support	**0**	**1.77**–**2.96**	**2.29**
Dialysis	**0**	**1.31**–**3.15**	**2.03**
Insurance			
Private	0.82	0.70–1.31	0.96
Public	**0.03**	**1.04**–**1.90**	**1.40**
Status 1	0.74	0.75–1.22	0.95
Region			
CA, NV, AZ, UT, NM	**<0.05**	**0.63**–**1.00**	**0.79**
AK, HI, ID, MT, OR, WA	**0.04**	**0.25**–**0.96**	**0.49**
Initial laboratory values			
Bilirubin	0.31	0.99–1.01	1.00
INR	0.41	0.97–1.08	1.02
Creatinine	0.09	0.95–1.81	1.31
Albumin	**0**	**0.70**–**0.89**	**0.78**

**Table 6:** Percent of patients who received a transplant, mean wait time to transplant, and intent-to-treat, waitlist, and post-transplant hazard ratios.

	Indication					
	Biliary atresia (reference group)	AHN	Metabolic disorders	Hepatoblastoma	Autoimmune cirrhosis	Other
Transplant within 1 yr of listing (%)	**51**	**59.8**	**72.6**	**85.1**	**47.6**	**50.8**
Transplant anytime within the study period (%)	**82.2**	**59.8**	**82.8**	**85.3**	**59.7**	**63.6**
Proportion of total study population (%)	27.6	13.2	11.2	5.6	2.6	39.8
Mean wait time (days)	111.2	94.1	115.8	23.6	386.8	198.2
Intent-to-treat hazard ratio	n/a	**1.78**	1.13	**3.71**	**1.87**	**2.15**
Waitlist hazard ratio	n/a	1.00	0.44	**6.82**	2.02	**3.48**
Post-transplant hazard ratio	n/a	**1.68**	1.07	**3.78**	**1.92**	**2.02**

## Data Availability

The data used to support the findings of this study are available from the Scientific Registry of Transplant Recipients at https://www.SRTR.org.

## Abbreviations

AHN: Acute hepatic necrosis
CI: Confidence interval
INR: International normalized ratio
PELD: Pediatric end-stage liver disease.
